# Function and Potential ceRNA Identification of Circ_009773 in Neodymium Oxide Nanoparticle-Induced Lung Epithelial Mesenchymal Transition

**DOI:** 10.3390/toxics12120917

**Published:** 2024-12-18

**Authors:** Lei Gao, Juan Juan, Zimeng Zheng, Lihua Huang

**Affiliations:** 1 Department of Public Health, International College, Krirk University, Bangkok 10220, Thailand; leilei_g@163.com; 2 School of Public Health, Baotou Medical College, Baotou 014030, China; j6834193381@163.com (J.J.); 15926217225@163.com (Z.Z.)

**Keywords:** NPs-Nd_2_O_3_, circ_009773, EMT, ceRNA

## Abstract

Nanoparticles of neodymium oxide (NPs-Nd_2_O_3_) can induce respiratory-related diseases, including lung tissue injury when entering the organism through the respiratory tract. However, it is currently unclear whether they can induce epithelial–mesenchymal transition (EMT) in lung tissue and the related mechanisms. In this study, we investigated the function of circ_009773 in the process of EMT induced by NPs-Nd_2_O_3_ in lung tissue from in vivo as well as in vitro experiments. The findings showed that NPs-Nd_2_O_3_ induced EMT in 16HBE cells and SD rat lung tissues. This was characterised by a decrease in epithelial markers and an increase in mesenchymal markers. Additionally, circ_009773 expression was found to decrease in 16HBE cells infected with NPs-Nd_2_O_3_ and also decreased in the lung tissues of SD rats. Relevant experiments showed that circ_009773 inhibited EMT in NPs-Nd_2_O_3_-treated 16HBE cells and SD rat lung tissues. The previous experiments revealed that circ_009773 was localised in the cytoplasm and functioned at the post-transcriptional level. With the EMT-related pathway used as the basis for circ_009773-related competing endogenous (ce)RNA mechanisms, our observations indicate that circ_009773 is capable of binding to and regulating the expression of miR-135b-5p. In summary, we found that circ_009773 inhibits the EMT induced by NPs-Nd_2_O_3_ in lung tissues, and this process likely occurs through competitive binding to miR-135b-5p.

## 1. Introduction

Neodymium oxide (Nd_2_O_3_) is a rare-earth material that has multiple applications. The demand for Nd_2_O_3_ is expanding due to the continuous development of modern industry, which poses unavoidable occupational health issues [1]. NPs-Nd_2_O_3_ have a columnar polyhedral structure, with an average dynamic size of about 50 nm. Our observations using transmission electron microscopy indicate that NPs-Nd_2_O_3_ can be endocytosed by 16HBE cells [2]. A multitude of in vivo and in vitro studies have demonstrated the increased risk of lung damage following exposure to Nd_2_O_3_ particles. Due to the small size of neodymium oxide nanoparticles (NPs-Nd_2_O_3_), they may have more serious biological health effects. A previous study conducted by our group confirmed that low-dose NP-Nd_2_O_3_ exposure induced inflammatory responses, oxidative stress, and DNA damage in human bronchial epithelial cells [2,3]. Epithelial–mesenchymal transition (EMT) is frequently observed in embryogenesis, organ fibrosis, and tumour metastasis and is linked with the pathogenesis of numerous diseases. Under normal conditions, epithelial cells are tightly bound to each other, have distinct polarity, and interact internally through intercellular junctions. However, in the course of EMT, the epithelial cells lose cellular junctions and their polarity. The process of EMT involves the upregulation of EMT mesenchymal cell markers, including Vimentin and α-smooth muscle actin (α-SMA), and the downregulation of epithelial cell markers, such as E-cadherin and ZO-1. Previous studies have found that neodymium oxide exposure induces lung fibrosis in animals [4]. While it is established that NPs-Nd_2_O_3_ can induce lung tissue injury, the specific mechanism by which they induce EMT in lung tissue injury remains unexplored. It is, therefore, important to explore their related functions.

A circular RNA is defined as a type of single-stranded RNA that forms a covalently closed loop. Unlike linear RNAs, circRNAs have linked 3′ and 5′ ends and lack a 5′ cap structure and a 3′ polyadenylate tail, rendering them insensitive to ribonucleases [5]. Due to their involvement in various biological processes, circRNAs have become a popular research topic in the RNA field. Numerous studies have been conducted on the functions of circRNAs. The primary and most common function of circRNAs is to act as sponges for specific tiny RNAs (miRNAs) and to regulate the stability and translation of mRNAs [6,7]. CircRNAs also play an important role in lung tissue injury; hsa_circ_0008305 was shown to be able to function in the EMT process by competing for the adsorption of miR-429/miR-200b-3p [8]. Moreover, in a related study on lung cancer, the knockdown of hsa_circ_000984 was identified as a potential inhibitor of the migration, invasion, proliferation, and EMT of NSCLC cell lines, after which it was experimentally demonstrated that circ_000984 exerts oncogenic effects through the regulation of Wnt/β-catenin pathway activation [9]. Our previous study found that the exposure of 16HBE cells to NPs-Nd_2_O_3_ can cause changes in the expression level of circ_009773; nevertheless, the role and underlying mechanism of the aberrant expression of circ_009773 in EMT induced by NPs-Nd_2_O_3_ in lung tissues currently remain unknown.

The present study focuses on elucidating the important function of circ_009773 in inducing EMT in lung tissues through exposure to NPs-Nd_2_O_3_, as well as predicting the network of competing endogenous mechanisms against it. There is a lack of population cohort studies on occupational exposure to rare earth elements, so this study provides experimental possibilities. At the same time, our results introduce a potential for the risk assessment of health hazards caused by NP-Nd_2_O_3_.

## 2. Materials and Methods

### 2.1. Cell Culture

Human bronchial epithelial cells (16HBE) were purchased from BLUEFBIO in Shanghai, China. The cells were cultured in medium (MEM; HyClone, Logan, UT, USA) supplemented with 10% foetal bovine serum (FBS; Four Seasons, Hangzhou, China) and 1% penicillin/streptomycin (Gibco, Gaithersburg, MD, USA). Human embryonic kidney cells (293-T), kindly supplied by Guangzhou Medical University, were cultivated in Dulbecco’s MEM (Biological Industries, Kibbutz Beit-Haemek, Israel) for a dual-luciferase reporter gene assay. The culture conditions were 37 °C and 5% CO_2_.

### 2.2. NP-Nd_2_O_3_ Treatment

NPs-Nd_2_O_3_ less than 100 nm in size were purchased from Sigma-Aldrich (St. Louis, MO, USA). To prepare for the subsequent experiments, 1 mg of NPs-Nd_2_O_3_ was dissolved in 1 mL of sterile phosphate-buffered solution. The mixed solution was sonicated for 30 min with a sonic device (Newtown, PA, USA) and then diluted with MEM to different concentrations (40, 80, and 160 μg/mL).

### 2.3. Animal Treatment

Forty-eight healthy male Sprague Dawley rats of SPF grade were selected with weights in the (200 ± 10 g) range (SIPEIFU Biotechnology Co., Ltd., Beijing, China). The rats were acclimatised for one week before being randomised into two groups: the NP-Nd_2_O_3_-dyed group (100 mg/kg) and the control group. The SD rats were treated via the intratracheal instillation method. The selection of the dose and method of contamination is based on the previous experience of the group [10]. The experimental groups received an NPs-Nd_2_O_3_ solution, while the control group received saline. An equivalent number of rats were euthanised on days 7, 14, and 28 following the treatment, respectively, and their lung tissues were collected for subsequent experimental studies. The Animal Ethics Committee of Baotou Medical College approved the experimental protocol used in this study (Ethics Approval No. 312: 2021004).

### 2.4. Cell Counting Kit-8 (CCK-8) Experiment

The 16HBE cells were treated with NPs-Nd_2_O_3_ at varying concentrations (0, 40, 80, and 160 μg/mL) for 48 and 72 h. Following this, the CCK-8 reagent (Tongren Institute of Chemical Research, Kyushu Island, Japan) was added, and the optical density values were measured at a wavelength of 450 nm.

### 2.5. qRT-PCR Analysis

RNA was successfully extracted from both 16HBE cells and lung tissue from the SD rats using TRIzol reagent (Invitrogen, Carlsbad, CA, USA). Cytoplasmic/nuclear RNA was extracted from 16HBE cells using an Ambion cytoplasmic/nuclear RNA extraction kit (Life Technologies, Carlsbad, CA, USA). The NanoDrop ND-2000 was utilised for the quantitative assessment of the RNA concentration (Thermo Science, Waltham, MA, USA). The extracted total RNA was reverse-transcribed into cDNA using the GoScript™ reverse transcription kit (Promega, Madison, WI, USA) using GoTaq^®^ qPCR Master Mix (Promega, Madison, WI, USA) on an ABI Verti PCR machine (ThermoFisher Scientific, Waltham, MA, USA) for real-time PCR. The analysis was carried out using the LightCycler Fluorescence Quantitative PCR system from Roche (Roche, Basel, Switzerland). The 2^−ΔΔCt^ method was used to calculate the expression levels of the relevant genes; this involved subtracting the CT-exposed group’s target gene value from its reference gene value and then subtracting the CT-control group’s target gene value from its reference gene value. All the primers utilised in this study were procured from Sangon Biotech (Shanghai, China), and the sequences can be found in Supplementary Table S1.

### 2.6. Western Blot (WB) Analysis

The total protein content of the samples was extracted using RIPA lysis buffer (Beyotime, Shanghai, China). The concentration was assayed by means of the Pierce BCA Protein Assay kit (Thermo Science, Rockford, IL, USA). Following denaturation, the protein was separated via SDS-PAGE and transferred onto a polyvinylidene difluoride (PVDF) membrane. After applying a sealing solution (Beyotime, Shanghai, China), the primary antibody and the membrane were incubated overnight, after which an incubation with the secondary antibody was carried out for an hour. The ECL luminescent solution was then applied for 1 min, and the membranes were immediately scanned using a two-colour infrared laser imaging system (Odyssey, LI-COR, Lincoln, NE, USA) and analysed for grey values. The antibodies used in this experiment were anti-E-cadherin (abcam, Cambridge, UK; ab1416, 212059), anti-β-actin (Abcam, Cambridge, UK; ab8226), and anti-α-smooth muscle actin (Affinity, Biosciences, Cincinnati, OH, USA; AF1032).

### 2.7. Cellular Transfection

IGE BIOTECHNOLOGY (Guangzhou, China) designed and synthesised two siRNAs targeting circ_009773 (Supplementary Table S2) and its negative control NC to inhibit its expression. Bersin Bio (Guangzhou, China) constructed the circ_009773 overexpression vector and its empty vector. Transfection was carried out by means of the Lipofectamine^®^ 2000 Transfection Kit (Invitrogen, Carlsbad, CA, USA).

### 2.8. Dual-Luciferase Reporter Gene Assay

The mutant and wild-type sequences of circ_009773 were inserted into the pmiRGlo vector. The vector was then co-transfected with an miRNA mimic (Supplementary Table S3) into 293-T cells. A fluorescence assay was carried out by means of the Dual-Luciferase^®^ Reporter Assay System Kit (Promega, Madison, WI, USA). The OD values of fluorophore fluorescence and sea kidney fluorescence of the samples were determined successively.

### 2.9. Statistical Analysis

Statistical analyses were performed using GraphPad Prism 8 (GraphPad Software, La Jolla, CA, USA) and SPSS 22.0 software (IBM Corporation, Armonk, NY, USA). The mean ± standard deviation was used to express all data. All experiments were replicated three times. The statistical methods used were the t-test and ANOVA. If *p* < 0.05, the difference was considered statistically significant.

## 3. Results

### 3.1. NPs-Nd_2_O_3_ Induces EMT in 16HBE Cells

**Figure 1:**
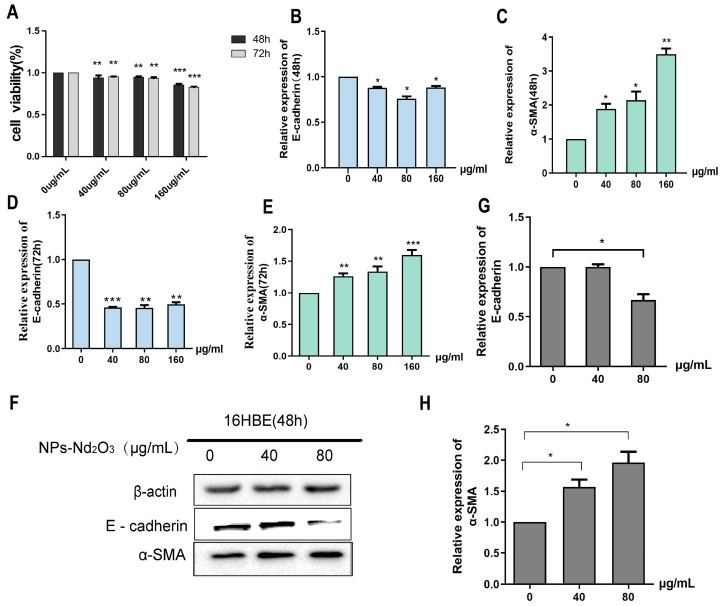
NPs-Nd_2_O_3_ induce EMT in 16HBE cells. (**A**) The cell survival of 16HBE cells treated with different doses of NPs-Nd_2_O_3_ was assessed using a CCK-8 assay. (**B**,**C**) The E-cadherin and α-SMA mRNA levels in 16HBE cells treated with different doses of NPs-Nd_2_O_3_ for 48 h using qRT-PCR assay. (**D**,**E**) The mRNA levels of E-cadherin and α-SMA measured in 16HBE cells treated with varying doses of NPs-Nd_2_O_3_ for 72 h using qRT-PCR assay. (**F**–**H**) The levels of E-cadherin and α-SMA proteins detected using a WB assay in 16HBE cells treated with different doses of NPs-Nd_2_O_3_ for 48 h. The data represented in the graphs are mean ± SD, *n* = 3, * *p* < 0.05, ** *p* < 0.01, *** *p* < 0.001 compared with 0 μg/mL group.

This study involved the exposure of 16HBE cells to a series of NP-Nd_2_O_3_ concentrations (0, 40, 80, 160 μg/mL). Cell viability was determined using the CCK-8 assay, which showed that the cell viability decreased with an increase in the exposure dose of NPs-Nd_2_O_3_ after 48 h and 72 h in human bronchial epithelial cells (Figure 1A). Next, the 16HBE cells were treated with 0, 40, 80, and 160 μg/mL of NPs-Nd_2_O_3_ for 48 and 72 h, and the expression levels of E-cadherin and α-SMA, markers of EMT occurrence, were determined. The results of the qRT-PCR assays showed that the expression level of E-cadherin was reduced and α-SMA expression was elevated in relation to the control group (Figure 1B–E). In summary, this evidence indicates that NPs-Nd_2_O_3_ exposure in human bronchial epithelial cells may induce epithelial-to-mesenchymal transition. Based on the above results and the cell survival limit, we utilised a Western blot assay with 16HBE cells exposed to NPs-Nd_2_O_3_ (0, 40, 80, 160 μg/mL) for 48 h for validation. The results of the Western blot experiments showed that after NPs-Nd_2_O_3_ exposure for 48 h, the E-cadherin level decreased to 80 μg/mL and α-SMA increased with increasing concentrations compared to the control group (Figure 1F–H). Therefore, the exposure of 16HBE cells to 80 μg/mL NPs-Nd_2_O_3_ for 48 h led to the most significant impact on epithelial–mesenchymal transformation, and the cell survival was greater than 80%. In the following functional experiments, the cells were exposed to 80 µg/mL NPs-Nd_2_O_3_ for 48 h.

### 3.2. NPs-Nd_2_O_3_ Exposure Induces EMT in Lung Tissue of Rat

**Figure 2:**
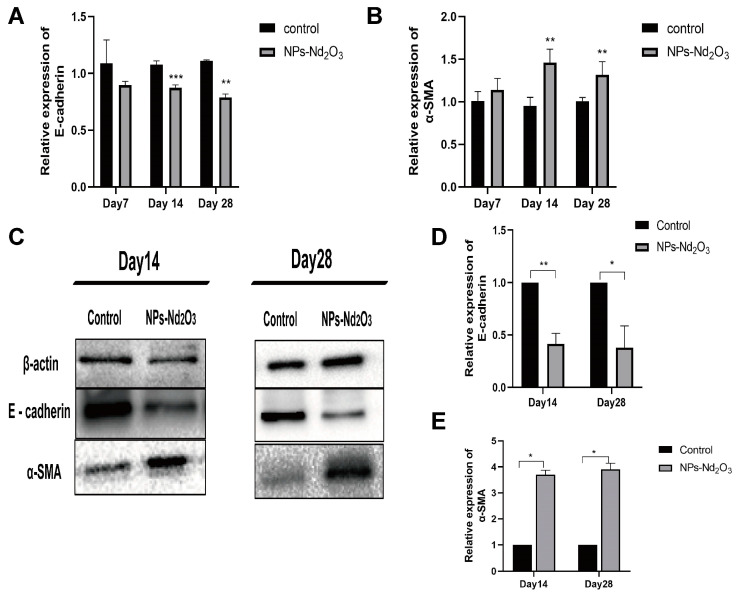
NPs-Nd_2_O_3_ exposure induces EMT in the lung tissue of rats. (**A**,**B**) qRT-PCR was used to detect the levels of E-cadherin and α-SMA mRNA in the rat lung tissues 7, 14, and 28 days after NPs-Nd_2_O_3_ treatment. (**C**–**E**) WB assay for E-cadherin and α-SMA protein levels in rat lung tissue after 14 and 28 days of NPs-Nd_2_O_3_ treatment. The data represented in the graphs are mean ± SD, *n* = 3, * *p* < 0.05, ** *p* < 0.01, *** *p* < 0.001 compared with the control group.

The previous experiments revealed that the exposure of SD rats to NPs-Nd_2_O_3_ resulted in the widening of the alveolar septa and the formation of incomplete alveolar structures in the 14- and 28-day exposure groups [10]. The occurrence of EMT in rat lung tissue was monitored at 7, 14, and 28 days following the intratracheal instillation of NPs-Nd_2_O_3_, and qRT-PCR assays were used to investigate the E-cadherin and α-SMA expressions. The results demonstrated a notable decrease in E-cadherin mRNA expression and a notable increase in α-SMA mRNA expression in the lung tissue of rats exposed to NPs-Nd_2_O_3_ for 14 and 28 days (Figure 2A,B). The qRT-PCR assays described above showed no significant difference in the expression of E-cadherin and α-SMA in the lung tissue of rats exposed to NPs-Nd_2_O_3_ for 7 days compared to the controls. Thus, the following WB experiments only observed the expression of E-cadherin and α-SMA proteins in the lung tissue of rats exposed to NPs-Nd_2_O_3_ for 14 and 28 days, and the results were consistent with those of the qRT-PCR assays (Figure 2C–E). The exposure to NPs-Nd_2_O_3_ induced significant changes in the lung epithelial–mesenchymal transformation indexes, indicating that neodymium oxide can induce EMT in lung epithelial cells.

### 3.3. circ_009773 Inhibition of NP-Nd_2_O_3_-Induced EMT in 16HBE Cells

**Figure 3:**
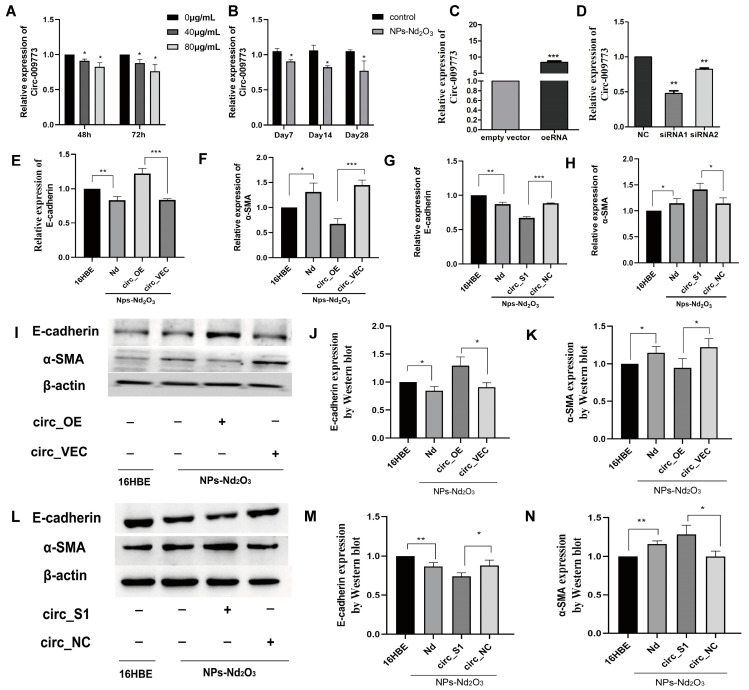
The circ_009773 inhibition of NP-Nd_2_O_3_-induced EMT in 16HBE cells. (**A**) The mRNA levels of circ_009773 in 16HBE cells treated with different doses of NPs-Nd_2_O_3_ were detected using qRT-PCR. (**B**) qRT-PCR experiments were conducted to detect the mRNA levels of circ_009773 in the lung tissues of NP-Nd_2_O_3_-treated rats. (**C**,**D**) qRT-PCR was used to detect the expression levels of circ_009773 after transfection with a circ_009773 OE vector and circ_009773 siRNA. (**E**,**F**) The mRNA levels of E-cadherin and α-SMA in 16HBE cells treated with NPs-Nd_2_O_3_ were detected using qRT-PCR assays after transfection with a circ_009773 OE vector. (**G**,**H**) After transfection with circ_009773 siRNA, qRT-PCR was used to detect the mRNA levels of E-cadherin and α-SMA in NP-Nd_2_O_3_-treated 16HBE cells. (**I**–**K**) The protein levels of E-cadherin and α-SMA in 16HBE cells treated with NPs-Nd_2_O_3_ were detected using WB assay after transfection with circ_009773 OE vector. (**L**–**N**) The protein levels of E-cadherin and α-SMA in NP-Nd_2_O_3_-treated 16HBE cells were detected using a WB assay after transfection with circ_009773 siRNA. The data represented in the graphs are mean ± SD, *n* = 3. Statistical significance was observed with * *p* < 0.05, ** *p* < 0.01, and *** *p* < 0.001 vs. 16HBE NC or VEC group.

circ_009773 was selected from the group’s pre-RNA-sequence results due to its significant differential expression. The outcomes of the present study showed that circ_009773 expression was significantly downregulated in 16HBE cells exposed to 80 μg/mL NPs-Nd_2_O_3_ for 48 h compared to the control group (Figure 3A). After the exposure of the rats to NPs-Nd_2_O_3_, the relative expression of circ_009773 was found to be decreased significantly in contrast to the control group (Figure 3B). These results suggest that a decrease in circ_009773 may be associated with the occurrence of corresponding lung tissue injury.

To elucidate the function of circ_009773 in NP-Nd_2_O_3_-induced EMT in 16HBE cells, experiments were conducted in which the effects of interfering with or overexpressing circ_009773 in 16HBE cells were examined. The expressions of E-cadherin and α-SMA, which are markers of epithelial-to-mesenchymal transition (EMT), were then analysed. A vector for overexpression (OE) and a vector for the blank plasmid control (VEC) were constructed for circ_009773 and transfected into 16HBE cells exposed to NPs-Nd_2_O_3_. The expression of circ_009773 was nearly 10-fold higher in the OE group compared to the VEC group (Figure 3C). Two siRNA sequences were designed and synthesised (Supplementary Table S2). The efficacy of the interference was assessed using a 16HBE cell model. The data indicated that both siRNA-1 and siRNA-2 were effective in downregulating circ_009773 expression. The interference efficiency of siRNA1 was 47.64%, which was more obvious than that of siRNA2 (Figure 3D), so siRNA1 was chosen to interfere with circ_009773 in the later experiments.

The qRT-PCR and WB assays showed that the overexpression of circ_009773 enhanced the expression level of E-cadherin and inhibited α-SMA expression (Figure 3E,F,I–K). The knockdown of circ_009773 by siRNA1 demonstrated that the downregulation of circ_009773 resulted in the repression of E-cadherin expression and enhanced the expression of α-SMA (Figure 3G,H,L–N). This research indicates that circ_009773 has an inhibitory effect on the epithelial-to-mesenchymal transition process induced in 16HBE cells by NPs-Nd_2_O_3_.

### 3.4. circ_009773 Binds to and Regulates miR-135b-5p Expression

**Figure 4:**
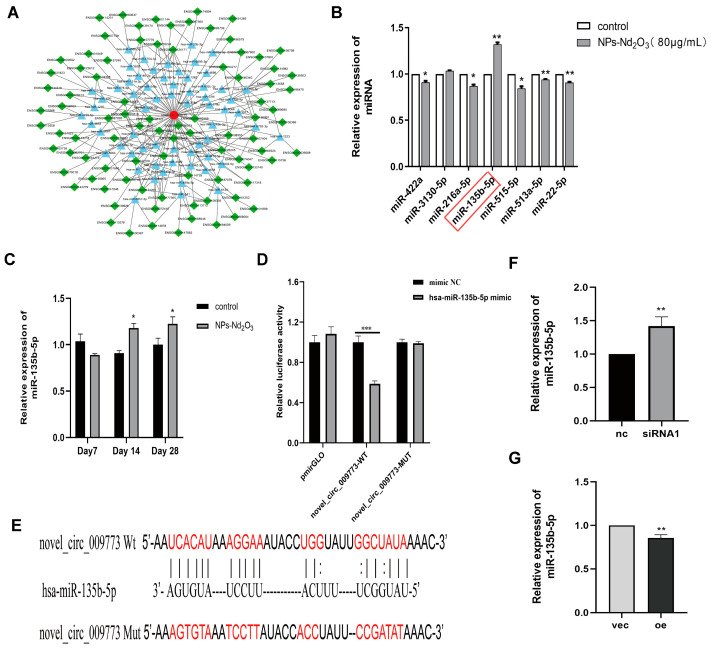
circ_009773 binds to miR-135b-5p and regulates its expression. (**A**) A co-regulated network consisting of circ_009773, 63 miRNAs, and 88 mRNAs determined via Cytoscape software (http://www.cytoscape.org/, accessed on 7 April 2023). (**B**) The control and infected cells expressed miR-135b-5p, miR-22-5p, miR-3130-5p, miR-422a, miR-216a-5p, miR-513a-5p, and miR-515-5p. (**C**) qRT-PCR was used to detect the levels of miR-135b-5p in the rat lung tissues after NP-Nd_2_O_3_ treatment. (**D**) The binding sites of the wild-type vector WT-has_circ_009773, mutant vector Mut-has_circ_009773, and miR-135b-5p mimic were identified. (**E**) A dual-luciferase reporter gene assay was conducted to detect luciferase activity. (**F**) The qRT-PCR assay detected a relative increase in miR-135b-5p expression following the overexpression of circ_009773. (**G**) The qRT-PCR assay detected a relative decrease in miR-135b-5p expression following interference with circ_009773. The data presented in the figure represent the mean ± SD of three independent experiments. Statistical significance is denoted as * *p* < 0.05, ** *p* < 0.01, and *** *p* < 0.001 compared with the control group.

Previous experiments confirmed that circ_009773 is predominantly located within the cytoplasm [3]. It is postulated that circ_009773 may be involved in post-transcriptional processes and acts as a “sponge” for miRNAs, which regulate the expression levels of target genes. Using the results of bioinformatics analysis, we selected 63 miRNAs and 88 mRNAs that interact with circ_009773 and are associated with EMT occurrence to map the co-regulatory network using Cytoscape 3.10.0 software (Figure 4A). Based on the literature search, we screened seven miRNAs involved in lung tissue damage processes, namely, miR-135b-5p [11,12], miR-22-5p [13], miR-3130-5p [14], miR-422a [15], miR-216a-5p [16], miR-513a-5p [17], and miR-515-5p [18]. A comparison between the control group and the experimental group revealed a difference in the expression of miR-135b-5p: it was found to be significantly elevated in 16HBE cells following exposure to NPs-Nd_2_O_3_ (Figure 4B). It was demonstrated that the expression of miR-135b-5p was markedly elevated in the lung tissues of SD rats exposed to NPs-Nd_2_O_3_ for 14 and 28 days in comparison to the controls (Figure 4C). These observations indicate that circ_009773 may compete for the adsorption of miR-135b-5p during the EMT process in 16HBE cells exposed to NPs-Nd_2_O_3_. The direct binding between circ_009773 and miR-135b-5p was successfully demonstrated; this outcome was validated through the use of a dual-luciferase reporter gene assay. The co-transfection of circ_009773-WT with an miR-135b-5p mimic resulted in a notable reduction in luciferase activity (Figure 4D,E). The findings of this study provide compelling evidence that circ_009773 can bind to miR-135b-5p. The expression of miR-135b-5p was found to be elevated after interfering with circ_009773 compared to the control group. Conversely, the overexpression of circ_009773 was found to result in a decrease in relative miR-135b-5p expression (Figure 4F,G). The results are an indication that circ_009773 may bind to miR-135b-5p and then become involved in the regulation of its expression.

## 4. Discussion

Neodymium oxide nanoparticles (NPs-Nd_2_O_3_) have been employed in a multitude of industrial applications to enhance the corrosion and abrasion resistance of metals. With large-scale mining and applications, there has been a noticeable increase in the incidence of environmental pollution and health hazards associated with NPs-Nd_2_O_3_. Previous experiments have demonstrated that respiratory system toxicity can be induced by exposure to NPs-Nd_2_O_3_ [2,3]. While our previous experiments revealed that LncRNA CNTFR-AS1 promotes Nd_2_O_3_ nanoparticle-induced DNA damage through homologous recombination repair [10], the mechanisms by which NPs-Nd_2_O_3_ cause EMT processes have rarely been investigated.

Epithelial–mesenchymal transition is a biological process whereby cells switch from an epithelial to a mesenchymal phenotype. EMT is involved in chronic inflammation, tissue remodelling, and a variety of fibrotic diseases and is closely associated with lung tissue injury [19,20,21]. It has been shown that nanoparticulate matter can enter cells and trigger inflammatory and fibrotic processes in the lungs and, in turn, induce processes involving EMT [21,22]. However, whether NPs-Nd_2_O_3_ induced EMT and its mechanisms in lung tissues was unclear. Epithelial cells are target organs susceptible to the toxic effects of environmental chemicals, and for this study, we selected human bronchial epithelial cells (16HBE). EMT is not a binary process but a heterogeneous and dynamically changing process with intermediate or partial characterisation states [23]. When epithelial–mesenchymal transition (EMT) occurs, the epithelial cells lose their polarity, and cellular connective structures are absent, while the markers of mesenchymal cells—fibronectin, Vimentin, and α-smooth muscle actin (α-SMA)—are upregulated. In contrast, epithelial cell markers such as E-cadherin and ZO-1 exhibit a reduction in expression [24]. In this study, we have selected the E-cadherin protein, which is the most characteristic manifestation of the epithelial–mesenchymal transition process, as the epithelial cell marker and α-SMA as the mesenchymal cell marker. It would be beneficial to incorporate more detailed protein data into subsequent comprehensive studies with the objective of more accurately defining EMT. The results of this study indicated that the proliferation of 16HBE cells is unaffected by exposure to 80 μg/mL NPs-Nd_2_O_3_ for 48 h. However, this process resulted in a reduction in E-cadherin expression and the upregulation of alpha-smooth muscle actin (α-SMA). Our findings demonstrated, for the first time, that exposure to NPs-Nd_2_O_3_ induces EMT in 16HBE cells.

In light of the fact that males are more often occupationally exposed to NPs-Nd_2_O_3_ in practical settings, male SD rats were selected for this study. A previous animal study revealed that instilling neodymium oxide into the bronchial tubes caused inflammation and fibrosis of the lungs in rats [25], while a study conducted by our research team demonstrated that DNA damage occurred in rat lung tissue from the intratracheal instillation of NPs-Nd_2_O_3_ [10]. It has been shown that rare-earth oxide nanoparticles can initiate EMT in rat lung tissue [26]. Therefore, it is important to investigate whether NPs-Nd_2_O_3_ can also cause EMT in SD rat lung tissues. In the present study, qRT-PCR assays confirmed that there was no significant change in the expression of EMT-related markers 7 days after NP-Nd_2_O_3_ exposure in the rats, which may be related to the fact that EMT is mainly involved in chronic inflammation and other chronic injury processes. Therefore, subsequent experiments were carried out that focused on rats exposed to NPs-Nd_2_O_3_ for 14 and 28 days. The results indicated that the expression of α-SMA increased incrementally in the lung tissue of the rats, while the expression of E-cadherin was found to decrease. Furthermore, this study aims to provide a scientific foundation for understanding the effects of chronic exposure to NPs-Nd_2_O_3_.

In recent years, due to the improvements in and application of bioinformatics analysis methods and high-throughput sequencing technologies, circRNAs have been recognised as being intimately connected with a multitude of human ailments, such as neurological disorders, cancer, and cardiovascular and cerebral vascular diseases [27,28,29]. Our group has previously shown that circRNAs are involved in the process of inflammation in NP-Nd_2_O_3_-induced 16HBE cells [30] and that circ_009773 plays certain functions in DNA damage induced by neodymium oxide nanoparticles [3]. It has also been shown that circRNAs play specific roles during the process of EMT in lung tissue; for example, the increased expression of circHECTD1 induces the involvement of EMT and its derivative fibroblast-like cells in lung fibrosis [31]. Thus, the objective of this study was to determine whether circ_009773 plays a role in inducing EMT in lung tissue caused by NPs-Nd_2_O_3_. The results indicate that circ_009773 was significantly downregulated in 16HBE cells treated with NPs-Nd_2_O_3_, and it was found to inhibit the occurrence of cellular EMT. Meanwhile, the expression of circ_009773 in the lung tissue samples from SD rats treated with NPs-Nd_2_O_3_ was found to be lower than that found in the control group. This study was based on the premise that circ_009773 has good homology between animals and humans.

It has been reported that circRNAs regulate gene expression at the post-transcriptional level by acting as miRNA “sponges” and competitively adsorbing miRNAs [5]. Through bioinformatics prediction and an analysis of the literature, we found that circ_009773 may competitively adsorb miR-135b-5p. We confirmed this through experiments such as dual-luciferase reporter gene assays and qRT-PCR. Although several other miRNAs are also involved in EMT and have binding sites with circ_009773, the differences are not significant.

Our findings indicated that circ_009773 directly bound to and regulated miR-135b-5p expression, which may play a role in NP-Nd_2_O_3_-induced EMT in lung tissues. Subsequently, based on this study, it will be possible to provide a more comprehensive perspective on the mechanistic study of EMT development in lung tissues due to NP-Nd_2_O_3_ exposure. Overall, we provide important evidence that circ_009773 is a key regulator of EMT in lung tissue induced by NP-Nd_2_O_3_ exposure.

## 5. Conclusions

To sum up, this study represents an inaugural investigation into the function of circ_009773 in NP-Nd_2_O_3_-induced EMT. circ_009773 was found to be poorly expressed in cells treated with NPs-Nd_2_O_3_ and rat lung tissues, thus inhibiting EMT development. A preliminary validation of the regulatory mechanism revealed that circ_009773 binds to miR-135b-5p and regulates its expression. The findings of this study demonstrate that circ_009773 plays a pivotal role in the EMT process induced by NPs-Nd_2_O_3_ in lung tissues.

## Data Availability

All data generated or analysed in this study are included in this published article.

## Supplementary Materials

The following supporting information can be downloaded at: https://www.mdpi.com/article/10.3390/toxics12120917/s1, Table S1. Primer sequences for qRT-PCR(5′ to 3′); Table S2. Sequences of siRNAs for circ-009773 knockdown; Table S3. Sequences of miR-135b-5p mimic.
