# Supplementary material for: Function and Potential ceRNA Identification of Circ_009773 in Neodymium Oxide Nanoparticle-Induced Lung Epithelial Mesenchymal Transition
Source: Toxics. 2024 Dec 18;12(12):917. doi: 10.3390/toxics12120917 (PMC11728544; doi:10.3390/toxics12120917)
Supplement: Supplementary file 1 [file toxics-12-00917-s001.zip › toxics-3330623-Supplementary Materials.pdf]

Supplementary Materials

Table S1.Primer sequences for *qRT-PCR*(5′ to 3′)

Gene	Forward primer	Reverse primer
β-actin	TTGTTACAGGAAGTCCCTTGCC	ATGCTATCACCTCCCCTGTGTG
E-cadherin	CGAGAGCTACACGTTCACGG	GGGTGTCGAGGGAAAAATAGG
α-SAM	TCCAAGTCTCTCAACAACTGGACT	CTGTCAGCAATGCCTGGGTA
Circ_009773	TGGTCGTGAGAGTCGTCAGTG	TCCAAGTCTCTCAACAACTGGACT

Table S2. Sequences of siRNAs for circ_009773 knockdown

Name	Sequences	
Circ_009773 siRNA1	Sense(5’to3’)	UAGGGACAGAAUUACUGGAAGdTdT
	Anti-sense(5’to3’)	CUUCCAGUAAUUCUGUCCCUAdTdT
Circ_009773 siRNA2	Sense(5’to3’)	AAUUAGGGACAGAAUUACUGGdTdT
	Anti-sense(5’to3’)	CCAGUAAUUCUGUCCCUAAUUAdTdT
Circ_009773 NC	Sense(5’to3’)	UUCUCCGAACGUGUCACGUdTdT
	Anti-sense(5’to3’)	ACGUGACACGUUCGGAGAAdTdT

Table S3. Sequences of miR-135b-5p mimic

Name	Sequences
miR-135b-5p	UAUGGCUUUUCAUUCCUAUGUGA
mimic NC	ACGUGACACGUUCGGAGAA
